# Supplementary material for: Development of a computer-aided design software for the quantitative evaluation of aesthetic damage
Source: PLoS One. 2019 Dec 18;14(12):e0226322. doi: 10.1371/journal.pone.0226322 (PMC6919621; doi:10.1371/journal.pone.0226322)
Supplement: S1 Code site — 1. (ZIP) [file pone.0226322.s001.zip › [EESC_jr][Projeto]Relatorio_07_21/vendors/select2/docs/_includes/options/core/data-attributes.html]

## Can I declare my configuration within the HTML?

It is recommended that you declare your configuration options for Select2
when initializing Select2. You can also define your configuration options
by using the HTML5 `data-*` attributes, which will override
any options set when initializing Select2 and any defaults.

### How should `camelCase` options be written?

HTML data attributes are case-insensitive, so any options which contain capital letters will be parsed as if they were all lowercase. Because Select2 has many options which are camelCase, where words are separated by uppercase letters, you must write these options out with dashes instead. So an option that would normally be called `allowClear` should instead be defined as `allow-clear`.

This means that if you declare your `<select>` tag as...

{% highlight html linenos %}
{% endhighlight %}

Will be interpreted the same as initializing Select2 as...

{% highlight js linenos %}
$("select").select2({
tags: "true",
placeholder: "Select an option",
allowClear: true
});
{% endhighlight %}

### Are options with nested configurations supported?

You can also define nested configurations, which are typically needed for
options such as AJAX. Each level of nesting should be separated by two
dashes (`--`) instead of one. Due to
a jQuery bug,
nested options using `data-*` attributes
do not work in jQuery 1.x.

{% highlight html linenos %}
{% endhighlight %}

Which will be interpreted the same as initializing Select2 with...

{% highlight js linenos %}
$("select").select2({
ajax: {
url: "http://example.org/api/test",
cache: true
}
});
{% endhighlight %}

The value of the option is subject to jQuery's
parsing rules for
HTML5 data attributes.
